# Prognosis and cardiotoxicity associated to adjuvant trastuzumab for breast cancer: real world study in a public health system

**DOI:** 10.61622/rbgo/2024rbgo93

**Published:** 2024-12-04

**Authors:** Ana Elisa Ribeiro da Silva Cabello, César Cabello, Susana Oliveira Botelho Ramalho, Otávio Rizzi Coelho, Otávio Rizzi Coelho-Filho, Helymar da Costa Machado, Délio Marques Conde, Luiz Carlos Zeferino

**Affiliations:** 1 Universidade Estadual de Campinas School of Medical Sciences Department of Obstetrics and Gynecology Campinas SP Brazil Department of Obstetrics and Gynecology, School of Medical Sciences, Universidade Estadual de Campinas, Campinas, SP, Brazil.; 2 Universidade Estadual de Campinas School of Medical Sciences Department of Internal Medicine Campinas SP Brazil Department of Internal Medicine, School of Medical Sciences, Universidade Estadual de Campinas, Campinas, SP, Brazil.; 3 Universidade Federal de Goiás Faculty of Medicine Department of Gynecology and Obstetrics Goiânia GO Brazil Department of Gynecology and Obstetrics, Faculty of Medicine, Universidade Federal de Goiás, Goiânia, GO, Brazil.

**Keywords:** Breast neoplasms, Cardiotoxicity, Chemotherapy, Prognosis, Trastuzumab, Unified Health System, Disease-free survival

## Abstract

**Objective:**

To analyze the prognosis of patients with breast cancer who developed trastuzumab-induced cardiotoxicity and to analyze factors associated with and resulting from cardiotoxicity.

**Methods:**

This was a retrospective cohort study that included 255 HER2-positive breast cancer patients who received adjuvant trastuzumab therapy. The inclusion criteria were a diagnosis of HER2-positive breast cancer and adjuvant trastuzumab therapy; disease stage I-III; <70 years; and a baseline echocardiogram showing a left ventricular ejection fraction (LVEF) ≥ 55%. The Kaplan-Meier method, the log-rank test, and the Cox proportional hazards model were used.

**Results:**

In all, 15.3% (39/255) of patients presented with cardiotoxicity. Treatment was suspended in 92.3% (36/39) of patients who presented with cardiotoxicity during trastuzumab treatment. The treatment was suspended in 46 of 255 patients and it was permanently interrupted in 84.8% (33/46) of these patients, with 84.8% (28/33) due to cardiotoxicity. Cardiotoxicity was not associated with disease-free survival (DFS) (hazard ratio (HR) = 1.48; 95% confidence interval (CI = 0.79-2.78) or overall survival (OS) (HR = 1.68; 95%CI= 0.83-3.41). Patients with clinical stage III and whom trastuzumab therapy was suspended (all causes) had worse DFS; (HR = 3.19; 95% CI=1.77-5.74) and (HR = 1.83; 95% CI=1.01-3.32) respectively. Those with clinical stage III and whom trastuzumab therapy was permanently interrupted had worse OS; (HR = 3.80; 95% CI =1.82-7.94), and (HR = 2,26; 95% CI =1.09-4.68 respectively.

**Conclusion:**

Cardiotoxicity was not associated with DFS or OS. Clinical stage III, Suspension and permanent interruption of treatment regardless of the cause were associated with worse DFS and OS in breast cancer patients.

## Introduction

The molecular subtypes of breast cancer have been incorporated into clinical practice to guide treatment decisions, and the expression of human epidermal growth factor receptor 2 (HER2) is an important component of this scenario. Epidermal growth factor receptors in humans, including HER1, HER2, HER3, and HER4, are transmembrane receptor tyrosine kinases with partial homology that regulate cell growth and survival as well as adhesion, migration, and differentiation.^(1)^ The HER2 gene is amplified in 25 to 30% of malignant breast tumors, and in these cases, the encoded protein is present at abnormally high levels in cells.^(2)^

Treatment for HER2-positive patients includes chemotherapy with anthracyclines and taxanes combined with anti-HER2 therapy.^(3)^ Cardiotoxicity is an adverse effect of the clinical treatment of breast cancer and is strongly related to the total cumulative dose (above 400 mg/m^2^) when induced by anthracyclines; cardiotoxicity can cause permanent damage to the myocardium through myocyte apoptosis, which results in fibrosis.^(4)^ Trastuzumab is related to the occurrence of heart failure in up to 26% of patients, and previous use of anthracyclines may favor the development of cardiotoxicity.^(5)^ The cardiodepressant effect mediated by trastuzumab is transient and reversible and most often manifests as an asymptomatic reduction in the left ventricular ejection fraction (LVEF) and less frequently as heart failure with functional limitations.^(6–8)^

The cardiotoxicity attributed to trastuzumab is still not well understood, but it is assumed to be partially due to blockade of HER2 receptors that are physiologically expressed on myocytes and that have essential cardiomyoprotective functions.^(9)^ Some risk factors, including advanced age, a high body mass index (BMI), and antihypertensive therapy, have been associated with cardiac dysfunction due to a reduced LVEF. Conversely, diabetes, heart valve disease, and coronary artery disease do not seem to significantly increase the risk of cardiac dysfunction.^(10–12)^

Trastuzumab therapy improves the prognosis of patients with HER2-positive breast cancer; thus, the causes and consequences of interrupting this treatment due to cardiotoxicity must be better understood to improve the clinical management of these patients.^(13)^ Therefore, the objectives of this study were to analyze the prognosis of breast cancer patients who developed cardiotoxicity induced by adjuvant trastuzumab therapy and to analyze factors associated with and resulting from cardiotoxicity.

## Methods

**Figure 1:**
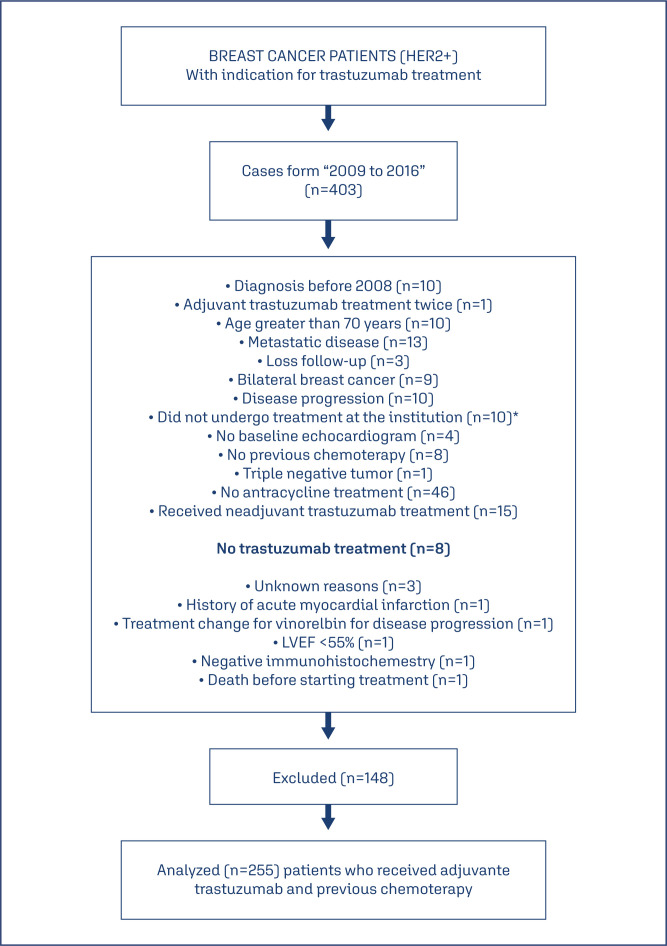
Patient selection process

In this retrospective cohort study, 403 medical records of consecutive patients treated between April 2009 and July 2016 at Dr. José Aristodemo Pinotti Women's Hospital/CAISM at the State University of Campinas (UNICAMP) and who were eligible for trastuzumab therapy were identified. After data collection, 148 patients were excluded for various reasons (Figure 1). Thus, 255 patients with HER2-positive breast cancer who received adjuvant trastuzumab therapy were included. The inclusion criteria were a diagnosis of HER2-positive breast cancer, stage I, II, III, who had in their treatment adjuvant chemotherapy containing trastuzumab, age less than 70 years; and a baseline echocardiogram showing an LVEF equal or above 55%, without symptoms or clinical manifestations of heart failure according to the Teichholz method. The exclusion criteria were treatment discontinuation for reasons unrelated to disease progression, other associated malignant neoplasms, pregnancy, and no history of undergoing a therapeutic regimen with anthracyclines. This manuscript has been prepared according to Strengthening the Reporting of Observational Studies in Epidemiology (STROBE) guidelines.

.*These patients were undergoing treatment at another hospital

The outcomes of this study were cardiotoxicity diagnosis during adjuvant trastuzumab therapy, disease-free survival (DFS), and overall survival (OS). It was considered as outcome to DFS, any recurrence of breast cancer; locoregional or systemic. Included contralateral breast cancer, and it was considered outcome to OS, mortality for any causes. Furthermore, we considered the beginning of trastuzumab treatment to the DFS and OS analysis. Echocardiograms were routinely performed at the same institution by experienced examiners. The following parameters were established for the diagnosis of cardiotoxicity: 1) cardiomyopathy with LVEF reduction, global or regional; 2) clinical symptoms and signs associated with heart failure; and 3) an LVEF reduction of at least 5% compared with the previous examination and an LVEF below 55% with concomitant signs or symptoms of heart failure, or an LVEF reduction of at least 10% compared with the previous examination and an LVEF below 55% without concomitant signs or symptoms.^(14)^ Clinical evaluations were scheduled every 45 days, and echocardiograms were scheduled every 90 days. When cardiotoxicity was diagnosed, trastuzumab treatment was suspended, which could be resumed pending a clinical evaluation by an oncologist. When clinical conditions were unfavorable, the treatment was permanently discontinued.

The adjuvant chemotherapy regimen for HER2-positive patients who also received trastuzumab therapy did not vary. All patients received adjuvant or neoadjuvant therapy with anthracycline combined with cyclophosphamide (CA), and 55.7% (142/255) received adjuvant or neoadjuvant therapy with anthracycline combined with cyclophosphamide followed by taxane (CA+T) (data not shown). The adjuvant trastuzumab therapy regimen was 8 mg/kg in the first cycle, followed by 6 mg/kg for a total of 17 cycles with an interval of 21 days. All infusions were prepared in 250-mL bags of 0.9% sodium chloride. The infusion time was 90 minutes for the first cycle and 30 minutes for the other cycles.

The following treatment information was analyzed: the cumulative anthracycline dose (corresponding to the sum of the doses of each cycle expressed in mg/m^2^), trastuzumab discontinuation due to cardiotoxicity, permanent trastuzumab discontinuation due to cardiotoxicity, and the interval between trastuzumab therapy initiation and death. The LVEF values of all echocardiograms were recorded. The follow-up time after the use of trastuzumab ranged from 12 to 96 months. Temporary suspension and permanent interruption of treatment for reasons other than cardiotoxicity were also analyzed.

HER2 testing was always performed in the same laboratory using immunohistochemistry (IHC), and the results were classified as no expression, 1+, 2+, or 3+. A result was considered positive when the expression was 3+. When the result was 2+, fluorescence in situ hybridization (FISH) was performed, and the result was considered HER2-positive when amplification of the respective gene was observed.^(2)^

Chi-square or Fisher's exact tests were used to compare categorical variables between groups, while the Mann-Whitney test was used to compare numerical variables between groups. The Kaplan-Meier method was used to construct DFS and OS curves, which were analyzed using the log-rank test. The Cox regression model was used to evaluate the prognostic factors associated with DFS and OS by univariate and multivariate analyses, and the results are expressed as unadjusted and adjusted hazard ratios (HRs), respectively. For the multivariate analyses of DFS and OS, the variables were included in the model as independent variables, and no variables were included as confounding variables. The data were analyzed using the statistical software SAS, version 9.2. The level of significance was set at 5%.

This study was approved by the Institutional Review Board of the School of Medical Sciences, State University of Campinas (UNICAMP) (protocol 1.413.577), under number: CAAE 45994915.3.0000.5404. This study was exempt from informed consent in accordance with the Declaration of Helsinki guidelines.

## Results

In all, 15.3% (39/255) of the patients presented cardiotoxicity during trastuzumab therapy. Among the 39 patients with cardiotoxicity, 32 had LVEF values below 55% in at least one echocardiogram during treatment, and the other seven patients had symptoms of heart failure without the LVEF decreasing below 55% (data not shown). The mean (standard deviation) ages of the women who presented and did not present with cardiotoxicity were 54.6 (± 9.8) and 50.8 (± 11.0) years (p=0.07), respectively. The frequency of cardiotoxicity in patients younger than 40 years was 4% (1/28), and in patients 40 years and older, the frequency was 16.7% (38/227) (p=0.09). Treatment was suspended in 92.3% (36/39) of patients with cardiotoxicity and was suspended in 4.6% (10/216) of patients for other reasons for a total of 46 cases of suspension (p<0.001). Treatment was permanently interrupted in 71.73% (33/46) of the patients whose treatment was initially suspended, including 71.8% (28/39) due to cardiotoxicity and 4.9% (11/222) for other reasons (p<0.001). Patient age, weight, BMI, hypertension, dyslipidemia, diabetes mellitus, smoking status, breast cancer laterality, and clinical stage were not associated with the diagnosis of cardiotoxicity (Table 1).

**Table 1:** Clinical and treatment characteristics of patients with breast cancer who received adjuvant trastuzumab therapy according to cardiotoxicity diagnosis

Clinical and treatment characteristics		Cardiotoxicity		p-value
		Yes	No	
		Mean ± SD	Mean ± SD	
Age (years)		54.6 ± 9.8	50.8 ± 11.0	0.07*
Weight (kg)		68.7 ± 12.4	70.2 ± 14.7	0.62*
Body mass index (kg/m^2^)		28.4 ± 5.1	27.8 ± 5.6	0.53*
Cumulative anthracycline dose (mg/m^2^)		299.3 ± 59.9	289.2 ± 64.9	0.49*
Number of cycles of trastuzumab given		10.8 ± 6.5	16.4 ± 2.5	< 0.001*
Treatment duration for trastuzumab		9.06 ± 6.5	12.2 ± 2.5	< 0.001*
		n(%)	n(%)	p-value
Dyslipidemia		3(7.7)	17(8.0)	1.00***
Diabetes mellitus		5(12.8)	22(10.2)	0.58***
Arterial hypertension		18(46.1)	77(35.6)	0.21**
Smoking status	Yes	8(20.5)	26(12.0)	0.29**
	No	26(66.7)	149(69.0)	
	Ex-smoker	5(12.8)	41(19.0)	
Age (years)	< 40	1(2.6)	27(12.5)	0.09***
	≥ 40	38(97.4)	189(87.5)	
Breast side	Right	16(41.0)	109(50.4)	0.27**
	Left	23(59.0)	107(49.5)	
Clinical stage	I	2(5.1)	17(7.8)	0.60**
	II	19(48.7)	88(40.7)	
	III	18(46.1)	111(51.4)	
Type of chemotherapy	Adjuvant	29(74.4)	130(60.7)	0.10**
	Neoadjuvant	10(25.6)	84(39.3)	
Radiotherapy	Yes	32(82.0)	198(92.0)	0.08***
	No	7(17.9)	18(8.3)	
Suspension of trastuzumab treatment	Yes	36(92.3)	10(4.6)	<0.001**
	No	3(7.7)	206(95.4)	
Permanent interruption of trastuzumab treatment	Yes	28(71.8)	5(2.3)	<0.001**
	No	11(28.2)	211(97.7)	
Total: distribution of patients		39(15.3)	216(84.7)	255(100)

* Mann-Whitney test;

** Chi-square test;

*** Fisher's exact test; SD = standard deviation

**Figure 2:**
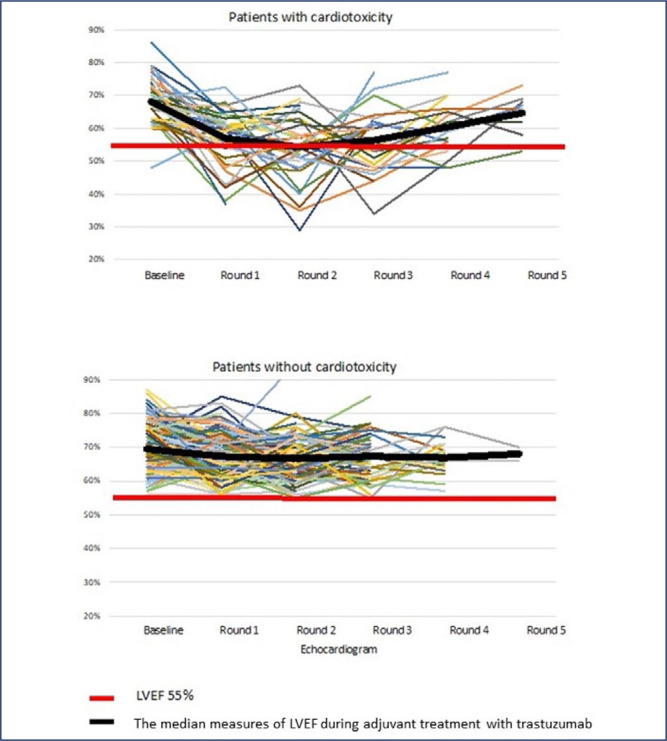
Left ventricular ejection fraction (LVEF) measures for patients with and without cardiotoxicity during adjuvant treatment with trastuzumab

Figure 2 illustrates the LVEF variation in patients with and without cardiotoxicity according to sequential echocardiogram results throughout adjuvant trastuzumab therapy. Most patients who developed cardiotoxicity showed a decrease in the LVEF in the first round of echocardiography after which they recovered, but treatment was suspended in most of those patients, as reported above.

**Figure 3:**
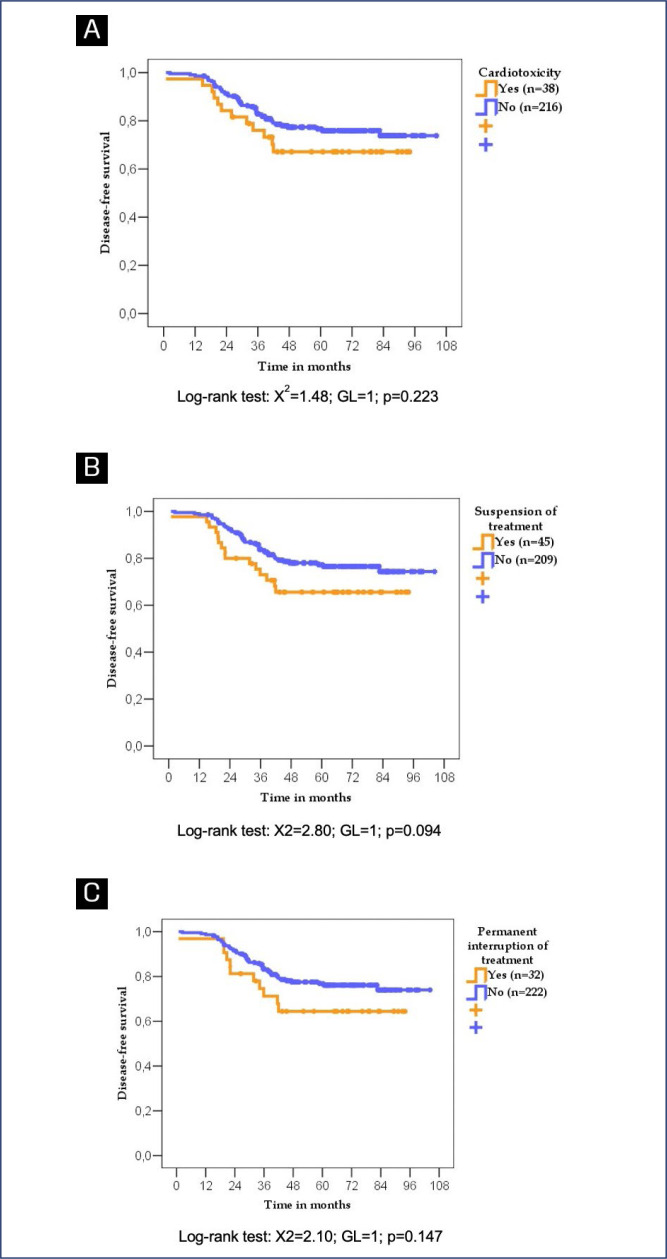
Disease-free survival of women with breast cancer according to cardiotoxicity diagnosis (A), treatment suspension due to any cause (B), and permanent treatment interruption due to any cause (C)

**Figure 4:**
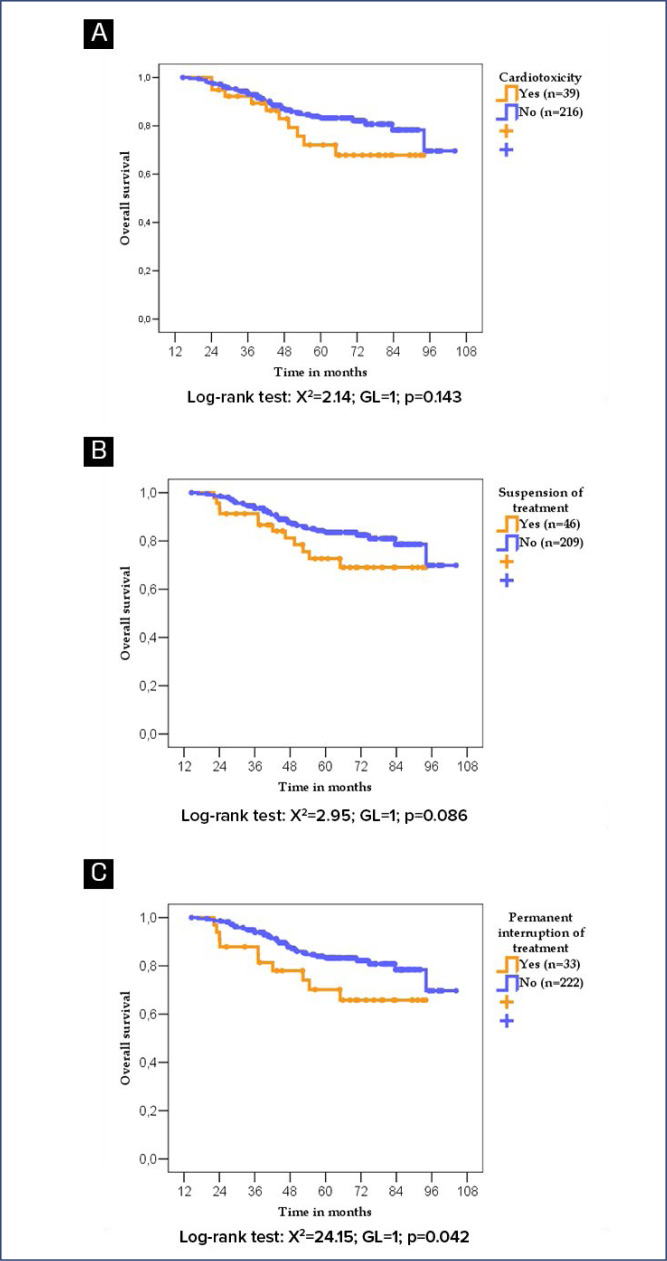
Overall survival of women with breast cancer according to cardiotoxicity diagnosis (A), treatment suspension due to any cause (B), and permanent treatment interruption due to any cause (C).

According to the log-rank test, patients who developed cardiotoxicity did not exhibit worse DFS or OS. Patients whose trastuzumab treatment was suspended or permanently interrupted due to cardiotoxicity or any other cause not exhibited worse DFS (p=0.094) and (p=0.147) respectively but worse OS when treatment was permanetly interrupted for any causes (p=0.042) (Figure 3 and 4).

Table 2 shows the results of the univariate and multivariate Cox regression analyses, which evaluated DFS. In the univariate analyses, patients with a BMI ≥ 33.1 kg/m^2^ presented (HR = 1.78; 95% confidence interval (CI) 0.98–3.24) and those younger than 40 years (HR = 1.47; 95% CI 0.70–3.10) respectively. Clinical stage III of breast cancer was the only associated to worse DFS. (HR = 3.27; 95% CI 1.83-5.86). Women who developed cardiotoxicity did not have worse DFS (HR = 1.48; 95% CI 0.79–2.78). The multivariate analysis showed that patients whose treatment was suspended for any cause (cardiotoxicity or any other cause) (HR = 1.83; 95% CI 1.01–3.32) and those with stage III breast cancer (HR = 3.19; 95% CI 1.77 –5.74) presented worse prognosis regarding to DFS.

**Table 2:** Disease-free survival of patients with breast cancer who received adjuvant trastuzumab therapy by univariate and multivariate Cox regression analyses

Clinical and treatment characteristics	Univariate analysis*			
	Categories	HR*	CI 95%*	p-value
Body mass index (kg/m^2^)***	< 33.11 (ref.)	1.00	---	---
	≥ 33.11	1.78	0.98 – 3.24	0.058
Age (years)	≥ 40 (ref.)	1.00	---	---
	< 40	1.47	0.70– 3.10	0.308
Arterial hypertension	No (ref.)	1.00	---	---
	Yes	0.67	0.39 – 1.16	0.152
Dyslipidemia	No (ref.)	1.00	---	---
	Yes	0.39	0.09 – 1.58	0.186
Diabetes mellitus	No (ref.)	1.00	---	---
	Yes	0.39	0.12 – 1.24	0.109
Smoking status	No (ref.)	1.00	---	---
	Yes	1.02	0.48 – 2.18	0.955
	Ex-smoker	1.09	0.58 – 2.08	0.786
Clinical stage	I+II (ref.)	1.00	---	---
	III	3.27	1.83 – 5.86	< 0.001
Type of prior chemotherapy	Adjuvant (ref.)	1.00	---	---
	Neoadjuvant	1.06	0.62 – 1.79	0.833
Radiotherapy	No (ref.)	1.00	---	---
	Yes	3.27	0.80 – 13.37	0.100
Breast side	Right (ref.)	1.00	---	---
	Left	1.26	0.76 – 2.09	0.374
Cumulative anthracycline dose (mg/m2) ***	< 359.6 (ref.)	1.00	---	---
	≥ 359.6	1.47	0.87 – 2.48	0.152
Any cardiotoxicity	No (ref.)	1.00	---	---
	Yes	1.48	0.79 – 2.78	0.227
Suspension of treatment due to any cause	No (ref.)	1.00	---	---
	Yes	1.64	0.91 – 2.93	0.098
Permanent interruption of treatment due to any cause	No (ref.)	1.00	---	---
	Yes	1.61	0.84 – 3.10	0.151
**Clinical and treatment characteristics**	Multivariate analysis**			
	**Categories**	HR*	CI 95%*	**p-value**
Clinical stage	I+II (ref.)	1.00	---	---
	III	3.19	1.77 – 5.74	< 0.001
Suspension of treatment due to any cause	No (ref.)	1.00	---	---
	Yes	1.83	1.01 – 3.32	< 0.046

* HR (hazard ratio) = ratio of risk for relapse; (n=193 censures and n=61 relapses). 95% CI HR = 95% confidence interval for the risk ratio. Ref.: reference level. (n = 254);

** HR (hazard ratio) = ratio of risk for relapse; (n = 193 censures and n = 59 relapses). 95% CI HR = 95% confidence interval for the risk ratio. Stepwise selection of variables. Ref.: reference level. (n = 252 total);

*** ROC Curve

Table 3 shows the results of the univariate and multivariate Cox regression analyses, which evaluated OS. In the univariate analyses, the patients with the worst OS were stage III breast cancer (HR = 3.93; 95% CI 1.89-8.16), breast side (HR = 1.89; 95% CI 1.03-3.48) and whose treatment was permanently interrupted due to any cause (HR = 2.05; 95% CI 1.01–4.15). Women who developed cardiotoxicity did not have worse OS (HR = 1.68; 95% CI 0.83–3.41). The multivariate analysis showed that patients whose treatment was permanently interrupted for any reason (cardiotoxicity or any other cause) (HR = 2.26; 95% CI 1.09–4.68), those with stage III breast cancer (HR = 3.80; 95% CI 1.82–7.67) exhibited worse OS.

**Table 3:** Overall survival of patients with breast cancer who received adjuvant trastuzumab therapy by univariate and multivariate Cox regression analyses

Clinical and treatment characteristics	Univariate analysis*			
	Categories	HR*	CI 95%	p-value
Age (years)	≥ 40 years (ref.)	1.00	---	---
	< 40 years	1.62	0.68 – 3.84	0.272
Body mass index (kg/m^2^) ***	< 33.11 (ref.)	1.00	---	---
	≥ 33.11	2.03	1.03 – 4.01	0.042
Arterial hypertension	No (ref.)	1.00	---	---
	Yes	0.69	0.36 – 1.33	0.270
Dyslipidemia	No (ref.)	1.00	---	---
	Yes	0.28	0.04 – 2.03	0.207
Diabetes mellitus	No (ref.)	1.00	---	---
	Yes	0.36	0.09 – 1.48	0.157
Smoking status	No (ref.)	1.00	---	---
	Yes	1.13	0.47 – 2.73	0.792
	Ex-smoker	1.59	0.81 – 3.15	0.180
Clinical stage	I+II (ref.)	1.00	---	---
	III	3.93	1.89 – 8.16	< 0.001
Type of chemotherapy	Adjuvant (ref.)	1.00	---	---
	Neoadjuvant	1.56	0.85 – 2.84	0.149
Radiotherapy	No (ref.)	1.00	---	---
	Yes	2.17	0.53 – 8.96	0.285
Breast side	Right (ref.)	1.00	---	---
	Left	1.89	1.03 – 3.48	0.041
Cumulative anthracycline dose (mg/m2) ***	< 359.71 (ref.)	1.00	---	---
	≥ 359.71	1.38	0.75 – 2.53	0.303
Any cardiotoxicity	No (ref.)	1.00	---	---
	Yes	1.68	0.83 – 3.41	0.148
Suspension of treatment due to any cause	No (ref.)	1.00	---	---
	Yes	1.78	0.91 – 3.45	0.090
Permanent interruption of treatment due to any cause	No (ref.)	1.00	---	---
	Yes	2.05	1.01 – 4.15	0.046
**Clinical and treatment characteristics**	Multivariate analysis**			
	**Categories**	HR*	**CI 95**%	**p-value**
Clinical stage	I+II (ref.)	1.00	---	---
	III	3.80	1.82 – 7.67	< 0.001
Permanent interruption of treatment due to any cause	No (ref.)	1.00	---	---
	Yes	2.26	1.09 – 4.68	0.028

* HR (hazard ratio) = risk ratio for death; (n=210 censures and n=45 deaths). 95% CI HR = 95% confidence interval for the risk ratio. Ref.: reference level. (n=255 total);

** HR (hazard ratio) = risk ratio for death; (n=210 censures and n=43 deaths). 95% CI HR = 95% confidence interval for the risk ratio. Stepwise selection of variables. Ref.: reference level. (n = 253 total)

## Discussion

This study showed that 15.3% of patients with HER2-positive breast carcinoma who received adjuvant trastuzumab therapy presented with cardiotoxicity, which resulted in treatment suspension and ultimately permanent treatment interruption in most of these cases. However, the study did not show that the diagnosis of cardiotoxicity, with or without suspension/permanent interruption of treatment, compromised the prognosis of patients.

When analyzing all suspensions or permanent interruptions of treatment, i.e., those due to causes other than cardiotoxicity, worse OS was observed. For every four treatment suspensions due to cardiotoxicity, at least one suspension occurred due to another cause. Patients whose trastuzumab treatment was interrupted received six fewer cycles on average than those whose treatment was not interrupted.

Sufficient evidence indicates that adjuvant trastuzumab therapy improves the prognosis of patients with HER2-positive breast cancer. A meta-analysis of clinical trials on the treatment of initial breast cancer showed that patients who received adjuvant trastuzumab therapy had lower mortality rates (relative risk [RR]: 0.66; 95% CI, 0.57-0.77), lower locoregional recurrence rates (RR, 0.58; 95% CI, 0.43-0.77), and lower distant recurrence rates (RR, 0.60; 95% CI, 0.52-0.78).^(15)^ A more recent and similar meta-analysis showed that patients with HER2-positive breast cancer exhibited better OS and had lower locoregional and distant recurrence rates (p = 0.001) with the addition of trastuzumab to their adjuvant therapy regimen.^(16)^ The addition of trastuzumab to their chemotherapy regimen resulted in a 37% improvement in OS (risk ratio [HR], 0.63; 95% CI, 0.54 to 0.73; p < 0.001) and an increase in the 10-year OS rate from 75.2% to 84%. These results were accompanied by an improvement of 40% in the DFS (HR, 0.60; 95% CI, 0.53 to 0.68; p < 0.001) and an increase in the 10-year DFS rate from 62.2% to 73.7%.^(17)^

One question to be considered is what minimum number of cycles or what length of adjuvant treatment offers some prognostic advantage to women. The FinHER study, which compared docetaxel *versus* vinorelbine for the adjuvant treatment of early-stage breast cancer, found that the subgroup of patients with HER2-positive breast cancer who received nine applications of trastuzumab had better recurrence-free survival after three years than those who did not receive the antibody (89% *versus* 78%; risk of recurrence or death, 0.42; 95% CI, 0.21-0.83).^(18)^ Another clinical trial that compared 6 months *versus* 12 months of adjuvant trastuzumab in patients with HER2-positive early breast cancer (PHARE) showed that after three to five years of follow-up, six months of trastuzumab therapy yielded worse outcomes than 12 months of therapy.^(19)^

Thus, the patients in our study whose treatment was suspended or permanently interrupted and who received an average of 11 cycles of trastuzumab may have experienced some benefit in disease control based on the FinHER study,^(18)^ but this benefit may be lower than would be expected with complete treatment based on the PHARE study.^(19)^ Another meta-analysis showed that, compared with short-term treatment, one year of trastuzumab prolonged OS and DFS in patients with HER2-positive early-stage breast cancer and that one year should be used as the standard of treatment.^(20)^

Our study sample cannot be considered representative of early-stage cancer since 52% (136/263) of the patients were diagnosed with stage III disease. Therefore, in this population, the impact of the treatment suspension or permanent interruption might be more harmful to the prognosis, even in studies with small sample sizes. In this period of time, at Brazilian public hospitals, the trastuzumab treatment would be suspended when the systemic progression was detected. Therefore, the prognosis of these patients may have been worsened by the negative effect of suspension or permanent interruption of trastuzumab therapy due to more advanced disease stage. In this study, multivariate Cox regression analyses showed that stage III disease and suspension or permanent interruption of treatment were associated with worse DFS and worse OS.

Kristeleit et al.^(21)^ reviewed long-term follow-up data on the efficacy, cardiac safety, and general safety of trastuzumab in several studies and confirmed a significant survival benefit with the addition of trastuzumab to the chemotherapy regimen in patients with HER2-positive disease; an acceptable safety profile was also noted. However, long-term cardiac safety data indicate the need for more frequent assessments in older patients.

At the time of this study, adjuvant treatment in the Brazilian healthcare system for patients with HER2-positive breast cancer included anthracyclines, followed by taxane and trastuzumab. The use of trastuzumab after the use of anthracyclines is associated with a higher probability of cardiotoxicity.^(22)^ In our study, all patients (n = 263) received previous anthracycline treatment. It is evident that clinical evaluation after treatment with anthracyclines and before the start of trastuzumab administration can identify patients already diagnosed with cardiotoxicity or heart failure, which prevents the occurrence of new cases with this adverse event. Furthermore, the cardiotoxicity caused by anthracycline use is associated with cumulative doses greater than 400 mg/m^2^.^(23)^ Our study showed that patients with or without cardiotoxicity received similar cumulative anthracycline doses of approximately 300 mg/m^2^.

This study presented some limitations. First aval, it was included locoregionally advanced carcinomas. To more consistently evaluate the exclusive effect of suspension or permanent interruption of trastuzumab treatment due to cardiotoxicity, a larger sample size of early breast carcinomas is necessary. It is acceptable to consider that these sample characteristics also limited more conclusive analyses of the association of other clinical morbidities. It is also possible to infer that for patients with HER2-positive breast carcinoma treated with trastuzumab, the possible deleterious effects of clinical morbidities, older age, and high BMI on the prevalence and severity of cardiotoxicity would be secondary to the cancer stage from a prognostic point of view. Finally, it is important to report the pertuzumab absence in our cases. The 6 years follow-up of the Aphinity clinical trial,^(24)^ confirmed an invasive disease-free survival (IDFS) benefice to node positive patients. Nevertheless, pertuzumab is not included in the Brazilian public health system to adjuvant treatment until nowadays. Although, the private clinics are allowed to use the trastuzumab plus pertuzumab schemes in our country, it represents less than 20% of population. Therefore, our data limited to a single HER2 blockage evaluation with trastuzumab, represents the reality of our clinic practice. And it is worthful of highlight. In addition, the trastuzumab plus chemotherapy based to taxanes without pertuzumab is a world standard of care to early breast cancer under 2cm of size and lymph nodes negative patients at public or private health system.^(25)^

In a similar study of Real world design, Lluch-Gómez et al.,^(26)^ observed 19.64% of general cardiotoxicity in 275 patients treated with tratuzumab plus chemotherapy, similar of our findings of 15.3% cardiotoxicity in 255 patients. Nevertheles, the Spanish study related a significant association of cardiotoxicity and overall survival (OR: 15.02 {7.437–30.335}). Independent of the number of trastuzumab cycles. These data are different of our finding. Based in our study, if the endpoint is survival, the impossibility of follow the ideal treatment is more important that the cardiotoxicity.

Thus, enabling treatment without permanent interruption of trastuzumab therapy is a desirable clinical target, and early and appropriate management of cardiotoxicity are important in improving prognosis.

## Conclusion

The cardiotoxicity related to trastuzumab adjuvant treatment was not associated to DFS and OS. The suspension or permanent interruption of the trastuzumab treatment and stage III, were associated to worst DFS and OS. Identifying patients at a higher risk of trastuzumab-induced cardiotoxicity, could prevent treatment interruptions. The clinical team should consider closer monitoring of the cardiac function of patients at a higher risk of cardiotoxicity.
